# Supplementary material for: The Early Pliocene extinction of the mega-toothed shark Otodus megalodon: a view from the eastern North Pacific
Source: PeerJ. 2019 Feb 13;7:e6088. doi: 10.7717/peerj.6088 (PMC6377595; doi:10.7717/peerj.6088)
Supplement: Supplemental Information 1 [file peerj-07-6088-s001.docx]

**Appendix 1. Stratigraphic justifications for *Otodus megalodon* occurrences used in the OLE analysis.**

**Locality:** San Juan Capistrano, California (USA)

**Formation:** Capistrano Formation, informal siltstone member

**Paleobiology Database #:** 152545

**Stratigraphic justification presented in PBDB entry:** yes

**Numerical Age reported by Pimiento and Clements (2014):** 11.6-3.6 Ma

**Corrected Age:** 5.6-3.7 Ma

**Comments:** An *Otodus megalodon* tooth (LACM 129982) mentioned in the Paleobiology Database entry for this locality was labeled as Blancan NALMA and assigned a late Miocene-early Pliocene age by Pimiento and Clements (2014). Diatoms from the horizon of SDNHM 53167 better constrain this age to 5.6-3.7 Ma (see main text).

**Locality:** Santa Cruz, California (USA)

**Formation:** Purisima Formation

**Paleobiology Database #:** 162439

**Stratigraphic justification presented in PBDB entry:** yes

**Numerical Age reported by Pimiento and Clements (2014):** N/A

**Corrected Age:** 6.9-5.3 Ma

**Comments:** This specimen was originally reported by Boessenecker (2016) and is summarized as 6.9-5.3 Ma (see main text).

**Locality:** La Joya, Mexico

**Formation:** San Diego Formation

**Paleobiology Database #:** 154115

**Stratigraphic justification presented in PBDB entry:** no

**Numerical Age reported by Pimiento and Clements (2014):** 3.6-2.6 Ma

**Corrected Age:** 4.2-3.6 Ma

**Comments:** SDNHM 29742 was collected from the base of the San Diego Formation. According to Vendrasco et al. (2012) the base of the San Diego Formation is as old as 4.2 Ma; typically reported as upper Pliocene. This stratum is lower in the San Diego Formation than a horizon that yielded Blancan NALMA correlative terrestrial mammals and magnetostratigraphic correlations indicating a minimum age of 3.6 Ma (Wagner et al., 2001). We therefore assign an age of 4.2-3.6 Ma to this occurrence.

**Locality:** Lawrence Canyon local fauna, California (USA)

**Formation:** San Mateo Formation, upper member

**Paleobiology Database #:** 50068

**Stratigraphic justification presented in PBDB entry:** yes

**Numerical Age reported by Pimiento and Clements (2014):** 10.3-4.9 Ma

**Corrected Age:** 5.33-4.6 Ma

**Comments:** Teeth of *Otodus megalodon* have been reported from both the San Luis Rey River local fauna (San Mateo Formation, lower unit) and the upper unit (Lawrence Canyon local fauna; Barnes et al., 1981; Domning and Deméré, 1984. Dates provided by Pimiento and Clements (2014) reflect the entire sequence at Oceanside, California, as interpreted by Domning and Deméré (1984), and do not match the Paleobiology Database entry. More precise dating is available for the upper unit of the San Mateo Formation which contains the Lawrence Canyon local fauna (see main text), summarized as 5.33-4.6 Ma.

**Locality:** Bolinas, California (USA)

**Formation:** Santa Cruz Mudstone

**Paleobiology Database #:** 152485

**Stratigraphic justification presented in PBDB entry:** no

**Numerical Age reported by Pimiento and Clements (2014):** 5.3-2.6 Ma

**Corrected Age:** 7.6-6.5 Ma

**Comments:** This record was originally reported as being derived from the Purisima Formation by Jordan and Hannibal (1923). The area was subsequently mapped by Galloway (1977) and Clark et al. (1984), the latter of which mapped most of the marine vertebrate-bearing exposures near Bolinas as the Santa Cruz Mudstone. We regard this as being the likely source of the “*Carcharodon branneri*” holotype specimen, and assign an age of 7.6-6.5 Ma based upon diatom biostratigraphy (see main text). Pimiento and Clements (2014) considered this an unreliable occurrence.

**Locality:** Garnet Canyon, California (USA)

**Formation:** Imperial Formation

**Paleobiology Database #:** 96541

**Stratigraphic justification presented in PBDB entry:** no (based on unpublished data)

**Numerical Age reported by Pimiento and Clements (2014):** N/A

**Corrected Age:** 6.43-4.187 Ma

**Comments:** This record from the Coyote Mountains of California was originally reported by Hanna (1926) as *Carcharodon arnoldi*, a junior synonym of *Otodus megalodon*. Magnetostratigraphic work on the nearby Fish Creek-Vallecito section of the Imperial Formation indicates an age of 6.43-4.187 Ma (Dorsey et al., 2011; see main text).

**Locality:** Elsmere Canyon, California (USA)

**Formation:** Towsley Formation

**Paleobiology Database #:** 97234

**Stratigraphic justification presented in PBDB entry:** yes

**Numerical Age reported by Pimiento and Clements (2014):** 5.3-3.6 Ma

**Corrected Age:** 10-5.3 Ma

**Comments:** Dates interpreted here from the stratigraphic literature and those reported by Pimiento and Clements (2014) do not overlap. Median Strontium dates from the base of the overlying Pico Formation range from 5.2-5.4 Ma (Beyer et al., 2009). A maximum age is provided by benthic foraminifera of the Delmontian stage (7.7.-5.2 Ma; Beyer et al., 2009), and Tortonian marine mammals (*Dusisiren jordani*, *Imagotaria downsi*) found in other deposits in California dated to 9-10 Ma indicate an age as old as 10 Ma (Whitmore and Barnes, 2008). Using the average of the two strontium dates for the Pico Formation, we assign an age range of 10-5.3 Ma (see Marx and Fordyce, 2015).

**Locality:** Kingsford Mine, Florida (USA)

**Formation:** Bone Valley Formation

**Paleobiology Database #:** 18548

**Stratigraphic justification presented in PBDB entry:** no

**Numerical Age reported by Pimiento and Clements (2014):** 10.3-4.9

**Corrected Age:** 10.3-4.9

**Comments:** Age determination by Pimiento and Clements (2014) is based upon correlation with the Hemphillian NALMA. Marine mammals collected from this mine were collected in association with Hemphillian land mammals from a stratigraphically higher source than the better-studied middle Miocene Bradley local fauna (Morgan, 1994). We agree with the age assessment of Pimiento and Clements (2014).

**Locality:** Payne Creek Mine, Florida (USA)

**Formation:** Bone Valley Formation

**Paleobiology Database #:** 18577

**Stratigraphic justification presented in PBDB entry:** yes

**Numerical Age reported by Pimiento and Clements (2014):** 5.3-3.6 Ma

**Corrected Age:** 10.3-4.9 Ma

**Comments:** The PBDB entry for this locality (10.3-4.9 Ma) does not match that reported by Pimiento and Clements (2014). Morgan (1994) correlates the unit with the latest Hemphillian (Hh4) which would indicate an age of 5.8-4.9 Ma; we conservatively use a Hemphillian correlation as for Kingsford Mine (see above).

**Locality:** Four Corners Mine (Palmetto fauna), Florida (USA)

**Formation:** Bone Valley Formation

**Paleobiology Database #:** 45478

**Stratigraphic justification presented in PBDB entry:** no

**Numerical Age reported by Pimiento and Clements (2014):** 5.3-3.6 Ma

**Corrected Age:** 5.8-4.9 Ma

**Comments:** The Palmetto fauna contains latest Hemphillian-correlative land mammals (Hh4; Morgan, 1994) and thus an age of 5.8-4.9 Ma is assignable (see Boessenecker et al., In Press).

**Locality:** East Coast Aggregates, Florida (USA)

**Formation:** Tamiami Formation

**Paleobiology Database #:** 152381

**Stratigraphic justification presented in PBDB entry:** no

**Numerical Age reported by Pimiento and Clements (2014):** 5.3-3.6 Ma

**Corrected Age:** 4.2-3.9

**Comments:** Williams et al. (2009) assigned an age of 4.2-3.9 Ma to the Tamiami Formation (see Boessenecker et al., in press). If from the very base of the Tamiami Formation, this occurrence could have an older maximum date (5.33 Ma).

**Locality:** Lee Creek Mine, North Carolina (USA)

**Formation:** Yorktown Formation

**Paleobiology Database #:** 52582

**Stratigraphic justification presented in PBDB entry:** yes

**Numerical Age reported by Pimiento and Clements (2014):** 5.3-3.6 Ma

**Corrected Age:** 4.9-3.92

**Comments:** A variety of dates are available for the basal Yorktown Formation (Sunken Meadow Member) including land mammal correlations (Tedford et al., 2004), strontium isotope ratios (Browning et al., 2009), radiometric dates from glauconite (Hazel, 1983), ostracods (Hazel, 1971), foraminifera and calcareous nannoplankton (Hazel, 1971; Dowsett and Wiggs, 1992). A maximum age is provided by 87Sr/86Sr and a minimum age is provided by calcareous nannoplankton, indicating an age of 4.9-3.92 Ma (see Boessenecker et al., in press, and Marx and Fordyce, 2015). Despite a plethora of published age determinations, Pimiento and Clements (2014) assigned an early Pliocene age without explanation.

**Locality:** Corkscrew Hill, Baja California Sur, Mexico

**Formation:** Tirabuzón Formation

**Paleobiology Database #:** 154117

**Stratigraphic justification presented in PBDB entry:** yes

**Numerical Age reported by Pimiento and Clements (2014):** 5.3-2.6 Ma

**Corrected Age:** 6.72-3.6 Ma

**Comments:** Dating is limited for the Tirabuzón Formation but the marine mammal fauna is consistent with an early Pliocene age and Barnes (1998) estimated the age to be 3-4 Ma. A tuff bed near the top of the underlying Boleo Formation has been dated at 6.76 Ma (Holt et al., 2000), providing a maximum age. Mollusks indicative of the early Pliocene have been reported from the overlying Infierno Formation, indicating the age of the Tirabuzón Formation is no younger than early Miocene. is Accordingly, we assign an age of 6.76-3.6 Ma to this occurrence (see main text).

**Locality:** Kambul, Yucatán, Mexico

**Formation:** Carrillo Puerto Formation

**Paleobiology Database #:** 58451

**Stratigraphic justification presented in PBDB entry:** no

**Numerical Age reported by Pimiento and Clements (2014):** Not included by Pimiento and Clements (2014), but included by Pimiento et al. (2015) as 10.3-4.6 Ma

**Corrected Age:** 10.3-4.6 Ma

**Comments:** Specimens of *Otodus megalodon* were reported by Domning (1989) who summarized the age as being correlative with the Hemphillian NALMA owing to land mammals from the same locality. Accordingly, Pimiento et al. (2015) assigned an age of 10.3-4.6 Ma, which we follow here.

**Locality:** Casa El Jebe, Falcón, Venezuela

**Formation:** Chiguaje Member, Codore Formation

**Paleobiology Database #:** 115791 (data from Carrillo-Briceno et al., 2015, not entered).

**Stratigraphic justification presented in PBDB entry:** no

**Numerical Age reported by Pimiento and Clements (2014):** N/A

**Corrected Age:** 5.33-3.6 Ma

**Comments:** Teeth of *Otodus megalodon* were reported from the Chiguaje Member of the Codore Formation by Carrillo-Briceno et al. (2015). The underlying El Jebe member of the Codore Formation spans the Miocene-Pliocene boundary, with a maximum age of 6 Ma (Smith et al., 2010). The minimum age of the formation was provided by planktonic foraminifera (*Globorotalia margaritae* and *Globorotalia* sp., cf. *G. miocenica*, which Smith et al. (2010) used to assign a minimum age of “middle Pliocene”. Aguirre-Fernandez et al. (2017) assigned an age range of 6-3 Ma for the formation as a whole. According to Wade et al. (2011), *Globorotalia margarita* has a FAD of 6.1 Ma and LAD of 3.9 Ma and *Globorotalia miocenica* has a FAD of 3.8 Ma and LAD of 2.4 Ma. Given the range of dates provided by these foraminifera, the tentative identification of a late Pliocene foraminifer, and the ‘middle Pliocene’ conclusion by Smith et al. (2010), we pick the Zanclean-Piacenzian boundary as the minimum age for this occurrence. The base of the Chiguaje member was interpreted as no older than Zanclean by Smith et al. (2010). We assign an age of 5.33-3.6 Ma for the Chiguaje Member of the Codore Formation.

**Locality:** El Yacural

**Formation:** Paraguana Formation

**Paleobiology Database #:** 107489

**Stratigraphic justification presented in PBDB entry:** yes

**Numerical Age reported by Pimiento and Clements (2014):** 5.33-3.6 Ma

**Corrected Age:** 5.33-3.6 Ma

**Comments:** Teeth of *Otodus megalodon* were reported from the El Hato Member of the Paraguana Formation by Aguilera et al. (2008). The age was reported without explanation as early Pliocene. An age of 5.33-3.6 Ma was assigned by Pimiento and Clements (2014) to this occurrence, which we follow here.

**Locality:** Punta la Colorada, Punta la Gorda

**Formation:** Onzole Formation

**Paleobiology Database #:** 151704, 151705

**Stratigraphic justification presented in PBDB entry:** yes

**Numerical Age reported by Pimiento and Clements (2014):** 5.33-2.6 Ma

**Corrected Age:** 5.33-3.4 Ma

**Comments:** Teeth of *Otodus megalodon* were originally reported from this unit by Longbottom (1979), who considered the Onzole Formation to be upper Miocene. The entry in the PBDB for these occurrences indicates a Pliocene age according to Aalto and Miller (1999), the apparent reasoning for the dates reported by Pimiento and Clements (2014). However, a Pliocene age is based on foraminifera reported by Whittaker (1988), who specifically identified foraminifera indicating zones N19-20, which correspond to an age of 5.3-3.4 Ma; therefore, we assign a more restricted age of 5.33-3.4 Ma to these two occurrences.

**Locality:** Punta Mansueto, Panama

**Formation:** Chagres Formation

**Paleobiology Database #:** 168112

**Stratigraphic justification presented in PBDB entry:** no (cites unpublished work)

**Numerical Age reported by Pimiento and Clements (2014):** N/A

**Corrected Age:** 8.29-5.12 Ma

**Comments:** Teeth of *Otodus megalodon* were reported from the Chagres Formation by Carrillo-Briceno et al. (2015). According to these authors (and references cited therein), the Chagres Formation is Messinian in age; however, in figure 1, the Chagres Formation is shown as corresponding to nannoplankton zones NN11 and NN12, indicating an age of 8.29-5.12 Ma.

**Locality:** Sunlands Pumping Station, South Australia, Australia

**Formation:** Loxton Sand

**Paleobiology Database #:** 50343

**Stratigraphic justification presented in PBDB entry:** no

**Numerical Age reported by Pimiento and Clements (2014):** 4.3-3.4 Ma

**Corrected Age:** 7.2-3.4 Ma

**Comments:** Shark teeth including *Otodus megalodon* were reported by Pledge (1985) who cited earlier work indicating correlation with the Kalimnan Stage (4.3-3.4 Ma). Later work by Miranda et al. (2009) indicates that deposition of the Loxton Sand initiated at 7.2 Ma and ceased by 3.4 Ma based upon strontium isotopes, and further stated that it is “likely that the youngest Loxton-Parilla Sands strandlines are older than approximately 5.0 Ma”. We conservatively assign an age of 7.2-3.4 Ma based upon the Kalimnan Stage correlation and Strontium ratios reported by Miranda et al. (2009).

**Locality:** Dutton Way, Victoria, Australia

**Formation:** Whaler’s Bluff Formation

**Paleobiology Database #:** 51328

**Stratigraphic justification presented in PBDB entry:** yes

**Numerical Age reported by Pimiento and Clements (2014):** 5.3-3.6

**Corrected Age:** 5.33-3.6

**Comments:** Teeth of *Otodus megalodon* were reported from this locality by Fitzgerald (2004, 2005). Deposition progressed through planktonic foraminiferal zones N19-21, though most of the unit corresponds to zones N19-20 (Fitzgerald, 2005, and references therein). Fitzgerald (2005) cites an age of 4.8-2.5 Ma. Stratigraphic position of specimens within the unit is unknown (Fitzgerald, 2005). Owing to uncertainties regarding the minimum age of the unit, we follow Pimiento and Clements (2014) in assigning an age of 5.3-3.6 Ma (early Pliocene), though a slightly younger maximum age may be defensible (Fitzgerald, 2005).

**Locality:** Beaumaris, Victoria, Australia

**Formation:** Black Rock Sand

**Paleobiology Database #:** 154112

**Stratigraphic justification presented in PBDB entry:** yes

**Numerical Age reported by Pimiento and Clements (2014):** 5.0-3.4 Ma

**Corrected Age:** 6.0-4.9 Ma

**Comments:** Teeth of *O. megalodon* were reported from the Black Rock Sand at Beaumaris by Fitzgerald (2004), who indicated a late Miocene-early Pliocene age (Cheltenhamian to Kalimnan stages). Based on the PBDB entry Pimiento and Clements (2014) used these stage boundaries for their age determination. However, more accurate Strontium isotope dates were available in the published literature (Dickinson et al., 2009) and cited in other publications by Fitzgerald (2012) and Fitzgerald et al. (2012). Strontium isotope dates indicate an age of 6.0-4.9 Ma (Dickinson et al., 2009), which we adopt here (see Fitzgerald and Kool, 2014).

**Locality:** Fossil Rock Stack, Victoria, Australia

**Formation:** Grange Burn Formation

**Paleobiology Database #:** 51335

**Stratigraphic justification presented in PBDB entry:** yes

**Numerical Age reported by Pimiento and Clements (2014):** 5.0-4.0 Ma

**Corrected Age:** 5.0-4.35 Ma

**Comments:** Teeth of *O. megalodon* were reported by Kemp (1991) and Fitzgerald (2004) from the Grange Burn Formation. Strontium isotope dates reported by Dickinson et al. (2002) indicate an age of 5.0-4.0 Ma, but the minimum date is further constrained by a K/Ar date of 4.35 Ma from overlying basalt (Turnbull et al., 1965). We therefore assign an age of 5.0-4.35 Ma, but cautiously note that it is unclear whether this K/Ar date has been recalculated using updated constants.

**Locality:** Pipiriki, Manawatu-Wanganui, New Zealand

**Formation:** Matemateaonga Sandstone

**Paleobiology Database #:** 152190

**Stratigraphic justification presented in PBDB entry:** no

**Numerical Age reported by Pimiento and Clements (2014):** 4.8-3.6 Ma

**Corrected Age:** 5.5-4.7 Ma

**Comments:** A tooth of *Otodus megalodon* was described by Keyes (1972; New Zealand Fossil Record Database number R21/f7495), although the provenience is poor: the specimen was purchased by the Wanganui Museum from a private collector in 1895 and the only locality information associated with the specimen noted it was collected from the town of Pipiriki. According to Ker (in Keyes, 1972), the matrix associated with the tooth resembles the Matemateaonga Sandstone. Pimiento and Clements (2014) presumably assigned an age of 4.8-3.6 Ma based upon the statement by Keyes (1972) that the unit corresponds to the Opoitian stage. More precise dating is available, however, and Vonk and Kamp (2004) indicated an age range of 5.5-4.7 Ma for the Matemateaonga Sandstone, and although they did not explain their reasoning, we assign these dates to this occurrence.

**Locality:** Patutahi Quarry, Gisborne, New Zealand

**Formation:** Tokomaru Formation

**Paleobiology Database #:** 155341

**Stratigraphic justification presented in PBDB entry:** no

**Numerical Age reported by Pimiento and Clements (2014):** N/A

**Corrected Age:** 7.2-3.7 Ma

**Comments:** A tooth of *Otodus megalodon* was described by Keyes (1972; New Zealand Fossil Record Database number N97/f0479) from Patutahi Quarry. Keyes (1972) did not indicate a stratum, only stating that the strata there were mapped as Opoitian-Mangapanian. Patutahi Quarry is located approximately 6 km west-southwest of Patutahi, NZ, and located at 38°38'14.15"S, 177°49'24.73"E. This quarry was mapped as the Opoitian-correlative Tokomaru Formation by Mazengarb and Speden (2000). The Opoitian stage ranges from 7.2-3.7 Ma, and we assign these dates to this occurrence.

**Locality:** Bonares – Casa del Pino, Huelva, Spain

**Formation:** Arenas de Huelva Formation

**Paleobiology Database #:** 151888

**Stratigraphic justification presented in PBDB entry:** yes

**Numerical Age reported by Pimiento and Clements (2014):** 5.33-3.6 Ma

**Corrected Age:** 5.33-3.6 Ma

**Comments:** Teeth of *Otodus megalodon* were reported by Garcia et al. (2009) from the Huelvas Sands. According to González-Delgado et al. (2004), the formation is early Pliocene in age. Because no further refined dates are available, we follow Pimiento and Clements (2014) in assigning an age of 5.33-3.6 Ma.

**Locality:** Can Picafort, Mallorca, Spain

**Formation:** Son Mir Sequence

**Paleobiology Database #:** 152233

**Stratigraphic justification presented in PBDB entry:** no (based on unpublished work)

**Numerical Age reported by Pimiento and Clements (2014):** 5.3-2.6 Ma

**Corrected Age:** 5.33-3.6 Ma

**Comments:** Teeth of *Otodus megalodon* were reported by Bauza Rullan (1948) from the Son Mir Sequence of Mallorca. Quoted text from the unpublished thesis of Marsili (2006) on the PBDB entry suggests a Pliocene age, yet Marsili (2006) continued to assign an early Pliocene age to these teeth based on their probable derivation from the Son Mir sequence, which is early Pliocene (Pomar et al., 1996). We follow Marsili (2006) in assigning an early Pliocene age to this occurrence (5.33-3.6 Ma).

**Locality:** Cré outcrop, Azores, Portugal

**Formation:** Touril Complex

**Paleobiology Database #:** 154113

**Stratigraphic justification presented in PBDB entry:** yes

**Numerical Age reported by Pimiento and Clements (2014):** 5.3-3.6 Ma

**Corrected Age:** 5.33-3.6 Ma

**Comments:** Teeth of *Otodus megalodon* were reported from the “Cré outcrop” by Avila et al. (2012). Based upon molluscan and foraminiferal biostratigraphy, the Touril Complex at Cré is firmly early Pliocene in age (Janssen et al., 2008); Pimiento and Clements (2014) therefore assigned an age of 5.3-3.6 Ma which we follow here.

**Locality:** Continental shelf, Portugal

**Formation:** unknown unit

**Paleobiology Database #:**

**Stratigraphic justification presented in PBDB entry:**

**Numerical Age reported by Pimiento and Clements (2014):** N/A

**Corrected Age:** 6.1-4.4 Ma

**Comments:** Teeth of *Otodus megalodon* were collected from the seafloor by fishing activities (Antunes et al., 2015). The teeth themselves have not been dated, but a number of cetacean specimens from the same deposit have been collected as well (Antunes et al., 2015). Sediment associated with these cetacean specimens have yielded a foraminiferal assemblage indicating an age of 6.1-4.4 Ma, which we assign to this occurrence.

**Locality:** Santa Margarida, Vale de Zebro, Portugal

**Formation:** Esbarronodadoiro Formation

**Paleobiology Database #:** N/A

**Stratigraphic justification presented in PBDB entry:** N/A

**Numerical Age reported by Pimiento and Clements (2014):** N/A

**Corrected Age:** 8.58-4.37

**Comments:** Teeth of *Otodus megalodon* were reported from these two localities by Antunes and Balbino (2003). According to Estevens (2000) this stratum corresponds to foraminiferal zones MN13-14 and N17-PL1; the latter indicate an age of 8.58-4.37 Ma.

**Locality:** Castell’Arquato, Miano, Colli Piacentini, Maiatico, Tra Lorenzana e Lari, Pienza, Siena, Colline Pisane

**Formation:** several

**Paleobiology Database #:** 152237, 152241, 152242, 152243, 152244, 152246, 152247, 152249

**Stratigraphic justification presented in PBDB entry:** yes

**Numerical Age reported by Pimiento and Clements (2014):** 5.33-2.6 Ma (Tra Lorenzana e Lari); 5.33-2.6 Ma (all others).

**Corrected Age:** 5.33-4.0 Ma

**Comments:** Most Pliocene Italian occurrences of *Otodus megalodon* in the Pimiento and Clements (2014) dataset were synthesized in the unpublished Ph.D. thesis of Marsili (2006), who reported some new occurrences and reviewed earlier reports. The record from Tra Lorenzana e Lari is early Pliocene according to Marsili (2006) and followed by Pimiento and Clements (2014). However, other localities which Marsili (2006) indicated were also early Pliocene (Maiatico) but assigned an early-late Pliocene age by Pimiento and Clements (2014). At least one of these localities (Castell’Arquato) has been mapped as early Pliocene in other studies (Bisconti, 2007, and references therein). Marsili (2006) considered all Mediterranean occurrences of *O. megalodon* to be early Pliocene at the youngest, and within the published literature, Marsili (2008) indicated that no specimens of *Otodus megalodon* date from rocks younger than 4 Ma. Therefore, we assign a minimum age of 4 Ma to these occurrences based upon Marsili (2008).

**Locality:** Matsukura Quarry, Towa-Sekizai, Ainosawa, and Myougane-Misaki, Chiba Prefecture, Japan

**Formation:** Senhata Formation

**Paleobiology Database #:** 70006 (Matsukura), 180528 (Towa-Sekizai)

**Stratigraphic justification presented in PBDB entry:** yes

**Numerical Age reported by Pimiento and Clements (2014):** N/A

**Corrected Age:** 6.3-5.12 Ma

**Comments:** Teeth of *Otodus megalodon* were reported with an extensive ichthyofauna from the Senhata Formation by Yabe and Hirayama (1998). Few age determinations come from the unit itself, but most researchers considered it to be latest Miocene to earliest Pliocene in age based upon planktonic foraminifera preserved in underlying, overlying, and coeval units (Kohno, 1992, and references therein). Kanie et al. (1991) correlated the Senhata Formation with calcareous nannoplankton zone CN10b, which has a minimum date of 5.12 Ma. A maximum age is provided by tephra beds; as interpreted by Murakami et al. (2016, and references therein), these indicate a maximum age of 6.3 Ma. We therefore assign an age of 6.3-5.12 Ma.

**Locality:** Kita-Daito-Jima Island, northeastern coast, Okinawa, Japan

**Formation:** Daito Limestone

**Paleobiology Database #:** 152404

**Stratigraphic justification presented in PBDB entry:** No

**Numerical Age reported by Pimiento and Clements (2014):** 3.6-0.8 Ma

**Corrected Age:** 4.7-3.3 Ma

**Comments:** Pimiento and Clements (2014) excluded this from their analysis; at least one other study (Yabe et al., 2004) regards this as one of the youngest occurrences of *Otodus megalodon* worldwide. A single tooth was reported by Yabe and Suguyima (1935) from a coralline limestone exposure of the Daito Limestone on the northeastern coast of Kita-Daito-Jima in the Borodino Islands. This unit is typically regarded as Pliocene-Pleistocene in age, but the maximum age of the formation is unknown and the entire unit was dolomitized beginning 2.0 Ma (Suzuki et al., 2006). The Daito Limestone is laterally heterogeneous as is to be expected for tropical reef carbonates, and Strontium isotope dates demonstrate that this unit has a complex depositional history, with dates ranging from 4.9-2.1 Ma (Takayanagi et al., 2010); the origin of the age range reported by Pimiento and Clements (2014) is unclear. However, strontium dates from the northeastern coastline of the island (samples 21-24, 26-27, 42-44) reported by Takayanagi et al. (2010) range in age from 4.7-3.3 Ma. We acknowledge that the exact locality is unclear, but because this entire portion of the island has yielded consistent dates we assign an age of 4.7-3.3 Ma to this occurrence.

**Locality:** Choshi area, Japan

**Formation:** Na-Arai Formation

**Paleobiology Database #:** unclear; perhaps 48094, 56942, or 181357

**Stratigraphic justification presented in PBDB entry:** no

**Numerical Age reported by Pimiento and Clements (2014):** N/A

**Corrected Age:** 5.33-4.36 Ma

**Comments:** Teeth of *Otodus megalodon* were reported by Itoigawa et al. (1975) from the Na-Arai Formation. Oishi and Hasegawa (1995) reviewed the stratigraphic literature for the Na-Arai Formation, concluding that it was early Pliocene; they also reported microfossils indicating a minimum age of 4.36 Ma. Owing to the early Pliocene age determinations in concert with this microfossil date, we assign an age of 5.33-4.36 Ma.

**Locality:** Sendai-Iwate area, Miyagi and Iwate Prefectures, Japan

**Formation:** Tatsunokuchi Formation

**Paleobiology Database #:** N/A

**Stratigraphic justification presented in PBDB entry:** N/A

**Numerical Age reported by Pimiento and Clements (2014):** N/A

**Corrected Age:** 5.6-3.9 Ma

**Comments:** Teeth of *Otodus megalodon* were reported from this unit by Oishi and Hasegawa (1995). The Tatsunokuchi Formation is typically reported as early Pliocene (Oishi and Hasegawa, 1995, and references therein). According to Yoshida et al. (2007, and references therein), the underlying Ishibane Formation has yielded a fission-track date of 5.6 Ma from a tuff bed, and the overlying Motohata Formation has yielded a fission-track date of 3.9 Ma. Though the age of the Tatsunokuchi Formation is likely narrower than this interval, we assign an age of 5.6-3.9 Ma to this occurrence.

**Appendix 2. Comments on *Otodus megalodon* occurrences excluded from the OLE analysis.**

**Locality:** Hawera

**Formation:** Tangahoe Formation

**Paleobiology Database #:** 28039

**Stratigraphic justification presented in PBDB entry:** no

**Numerical Age reported by Pimiento and Clements (2014):** 3.6-3.0 Ma

**Comments:** The citation on the PBDB entry for this faunal assemblage is McKee (1987), which does not mention anything about *Otodus megalodon*. A single conference abstract (McKee, 1994A) reports vertebrae of *O. megalodon* from the Tangahoe Formation at Hawera. Extensive collections have been made from this locality (McKee, 1994B) but virtually all of these fossils reside in a private collection. We excluded this occurrence because these fossils are in a private collection and cannot be studied at present. Accordingly, this occurrence has not been given a New Zealand Fossil Record Database entry.

**Locality:** Wanganui-Taranaki

**Formation:** Unknown

**Paleobiology Database #:** 152191

**Stratigraphic justification presented in PBDB entry:** no

**Numerical Age reported by Pimiento and Clements (2014):** 5.3-0.3 Ma

**Comments:** This specimen of *Otodus megalodon* was reported by Keyes (1972), who indicated that the locality was initially confused between New Zealand and Australia, and that the original label indicated the specimen was “probably from the upper Miocene beds Older Wanganui Series of NZ Geological Survey from between Wanganui and N. Plymouth”. New Plymouth and Whanganui are separated by approximately 180 km of coastline, at least half of which consists of Miocene-Pleistocene marine rocks. We excluded this record from our analysis because of the poor provenience of this specimen.

**Locality:** Cameron Inlet, Tasmania, Australia

**Formation:** Cameron Inlet Formation

**Paleobiology Database #:** 51414

**Stratigraphic justification presented in PBDB entry:** yes

**Numerical Age reported by Pimiento and Clements (2014):** 5.3-2.6 Ma

**Comments:** A tooth of *Otodus megalodon* was reported by Kemp (1991: Plate 30C). The root is not preserved, and this specimen has coarse serrations, indicating this is most likely a misidentified *Carcharodon carcharias*. Because it does not appear to be an *Otodus megalodon* tooth, we do not include it.

**Locality:** Farol das Lagostas, Angola

**Formation:** Luanda Formation

**Paleobiology Database #:** 154111

**Stratigraphic justification presented in PBDB entry:** yes

**Numerical Age reported by Pimiento and Clements (2014):** 5.3-2.6 Ma

**Comments:** Teeth of *Otodus megalodon* were reported by Antunes (1978). The age of the unit has been considered Miocene or Pliocene and as old as late early Miocene; according to Antunes (1978), the unit is Pliocene in age based upon the shark assemblage. However, most modern studies consider the unit entirely Miocene (Cauxeiro et al., 2014, and references therein). Because of the conflicting age determinations and lack of sub-epoch age control within the Pliocene, we have excluded this record from our dataset. Furthermore, we note that the age determination is based upon the shark assemblage, based principally off the co-occurrence of *Carcharodon carcharias* and *Otodus megalodon*; using this date in a dataset to evaluate the extinction date for *O. megalodon* is a case of circular reasoning.

**Locality:** Main Vertebrate Spot, Libya

**Formation:** unknown

**Paleobiology Database #:** 136597

**Stratigraphic justification presented in PBDB entry:** no

**Numerical Age reported by Pimiento and Clements (2014):** 5.3-3.6 Ma

**Comments:** The stratigraphic assessment and shark fauna, including *Otodus megalodon*, were reported by Pawellek et al. (2012). The age determination as early Pliocene was based upon the co-occurrence of *Otodus megalodon* and *Carcharodon carcharias*. Because the minimum age of the locality is based upon *Otodus* *megalodon*, including it within our dataset (e.g. Pimiento and Clements, 2014) is a case of circular reasoning; we therefore exclude it from our dataset.

**Locality:** Fort Green Mine SW, North Palmetto Mine, Achan Mine, Palmetto Mine (Agrico), Chicora Mine

**Formation:** Bone Valley Formation

**Paleobiology Database #:** 151641, 151679, 151680, 151681, 151682 (respectively)

**Stratigraphic justification presented in PBDB entry:** no

**Numerical Age reported by Pimiento and Clements (2014):** 5.3-3.6 Ma

**Comments:** These five occurrences were included in the Pimiento and Clements (2014) dataset. According to the PBDB entries for all five, “Locality based on FLMNH online data base.” No further stratigraphic justifications are provided in the PBDB entry or on the Florida Museum of Natural History database, nor are they provided in the supplementary tables of Pimiento and Clements (2014). Though the age assignment by Pimiento and Clements (2014) is reasonable based upon other assemblages from the upper Bone Valley Formation, ultimately the unpublished stratigraphic framework cannot be vetted at present and these occurrences are excluded from our dataset.

**Locality:** Bahia Inglesa, Chile

**Formation:** Bahia Inglesa Formation

**Paleobiology Database #:** 154114

**Stratigraphic justification presented in PBDB entry:** yes

**Numerical Age reported by Pimiento and Clements (2014):** 16.0-3.6 Ma

**Comments:** Shark teeth from Bahia Inglesa were originally reported by Long (1993) and an early Pliocene age was assigned based upon diatoms indicating an age of 4.5-2.6 Ma (Tsuchi et al., 1988). This occurrence was later identified as the Lechero Member of the Bahia Inglesa Formation (Walsh and Suarez, 2006); however, these specimens were provided to D.J. Long without intraformational stratigraphic control, so attribution to the Lechero Member is not possible and should be disregarded. The maximum age of fossiliferous strata below the Bahia Inglesa Bonebed (=Morro Member) is unclear but typically reported as Miocene (Walsh and Suarez, 2006). *Otodus megalodon* teeth originally reported by Long (1993) were not reported to a specific stratigraphic horizon, and the dates provided by Pimiento and Clements (2014) are for the entire sequence. The only specimens of *O. megalodon* reported with stratigraphic position indicated were reported in an unpublished thesis (Walsh, 2001) from the Bahia Inglesa Bonebed, which is older than the 7.6 Ma old ash bed and not informative for this analysis. Conflicting age determinations, unsatisfactory provenience, and an entirely late Miocene age for specimens with provenience lead us to exclude this occurrence from the dataset.

**Locality:** Darby Sink, Antigua and Barbuda

**Formation:** Highlands Formation

**Paleobiology Database #:** 151880

**Stratigraphic justification presented in PBDB entry:** no

**Numerical Age reported by Pimiento and Clements (2014):** 5.3-2.6 Ma

**Comments:** A partial tooth of *Otodus megalodon* was reported by Flemming and McFarlane (1998) from an exposure of the Highlands Formation in Darby Sink, a large karstic sinkhole. These authors cite a Pliocene age indicated by Watters et al. (1992), though Watters et al. do not cite any evidence in favor of a Pliocene age, in turn citing Brasier and Mather (1975). However, Brasier and Mather (1975) actually reported microfossils which indicate a minimum age of late Miocene, and based upon a synthesis of diagenetic data and microfossils these authors concluded the Highlands Formation is middle Miocene in age. Therefore, the occurrence is too old to be informative and is excluded from the dataset.

**Locality:** Loma Phinney, Cuba

**Formation:** Canímar Formation

**Paleobiology Database #:** 151881

**Stratigraphic justification presented in PBDB entry:** no

**Numerical Age reported by Pimiento and Clements (2014):** 11.6-2.6 Ma

**Comments:** Teeth of *Otodus megalodon* were reported from the Canímar Formation by Iturralde-Vinent et al. (1996), who assigned an age of late Miocene to Pliocene. A Pliocene or even Pleistocene age is indicated by some studies (Franco, 1992). Because of the lack of subepochal age control for this occurrence, it is excluded from the dataset.

**Locality:** Rancho Algodones, Baja California Sur, Mexico

**Formation:** Salada Formation

**Paleobiology Database #:** 154118

**Stratigraphic justification presented in PBDB entry:** yes

**Numerical Age reported by Pimiento and Clements (2014):** 5.3-2.6 Ma

**Comments:** A shark assemblage including *Otodus megalodon* was reported by Espinosa-Arrubarrena and Applegate (1981). The Pliocene age of the locality was based upon the elasmobranch assemblage. No biostratigraphically informative microfossils or ash dates have been reported, with different authors proposing one or all sections of the Salada Formation being early-late Pliocene or early Pliocene in age (see Martinez-Gutierrez and Sethi, 1997, and references therein). Owing to the imprecise minimum age control and the lack of subepochal age determinations, this occurrence is excluded from our dataset.

**References**

Aalto KR, and Miller W. 1999. Sedimentology of the Pliocene upper Onzole Formation, an inner-trench slope succession in northwestern Ecuador. *Journal of South American Earth Sciences* 12:68-85.

Aguilera OA, Garcia L, and Cozzuol MA. 2008. Giant-toothed white sharks and cetacean trophic interaction from the Pliocene Caribbean Paraguana Formation.

Aguirre-Fernandez G, Mennecart B, Sánchez-Villagra MR, Sánchez R, and Costeur L. 2017. A dolphin fossil ear bone from the northern Neotropics-insights into habitat transitions in iniid evolution. *Journal of Vertebrate Paleontology* 37:e1315817.

Antunes MT. 1978. Faunes ichthyologiques du Néogène supérieur d'Angola, leur age, remarques sur le Pliocéne marin en Afrique australe. *Ciênces da Terra (UNL)* 4:59-90.

Antunes MT, and Balbino A. 2003. Uppermost Miocene lamniform selachians (Pisces) from the Alvalade basin (Portugal). *Ciencias da Terra (UNL)* 15:141-154.

Antunes MT, Legoinha P, and Balbino A. 2015. Megalodon, mako shark and planktonic foraminifera from the continental shelf off Portugal and their age. *Geologica Acta* 13:181-190.

Avila SP, Ramalho R, and Vullo R. 2012. Systematics, palaeoecology and palaeobiogeography of the Neogene fossil sharks from the Azores (Northeast Atlantic). *Annales de Paleontologie* 98:167-189.

Barnes LG. 1998. The sequence of fossil marine mammal assemblages in Mexico. *Avances en Investigacion: Paleontologia de Vertebrados, publicacion especial* 1:26-79.

Barnes LG, Howard H, Hutchison JH, and Welton BJ. 1981. The vertebrate fossils of the marine Cenozoic San Mateo Formation at Oceanside, California. In: Abbott PL, and O'Dunn S, eds. *Geologic Investigations of the coastal plain, San Diego County, California*. San Diego, California: San Diego Association of Geologists, 53-70.

Bauzá Rullán J. 1948. Nuevas aportaciones al conocimiento de la ictiologia del Neógeno Caralano-Balear. *Estudios Geológicos* 8:1-19.

Beyer LA, McCulloh TH, Denison RE, Morin RW, Enrico RJ, Barron JA, and Fleck RJ. 2009. Post-Miocene right separation on the San Gabriel and Vasquez Creek Faults, with supporting chronostratigraphy, western San Gabriel Mountains, California. *US Geological Survey Professional Paper* 1759:1-43.

Bisconti M. 2007. A new basal balaenopterid whale from the Pliocene of northern Italy. *Palaeontology* 50:1103-1122.

Boessenecker RW. 2016. First record of the megatoothed shark *Carcharocles megalodon* from the Mio-Pliocene Purisima Formation of Northern California. *PaleoBios* 33.

Brasier M, and Donahue H. 1975. The stratigraphy of Barbuda, West Indies. *Geological Magazine* 112:271-282.

Browning JV, Miller KG, Mclaughlin PP, Edwards LE, Kulpecz AA, Powars DS, Wade BS, Feigenson MD, and Wright JD. 2009. Integrated sequence stratigaphy of the postimpact sediments from the Eyreville core holes, Chesapeake Bay impact structure inner basin. *Geological Society of America Special Paper* 458:775-810.

Carrillo-Briceño JD, Gracia Cd, Pimiento C, Aguilera OA, Kindlimann R, Santamarina P, and Jaramillo C. 2015a. A new late Miocene chondrichthyan assemblage from the Chagres Formation, Panama. *Journal of South American Earth Sciences* 60:56-70.

Carrillo-Briceño JD, Maxwell E, Aguilera OA, Sánchez R, and Sánchez-Villagra MR. 2015b. Sawfishes and other elasmobranch assemblages from the Mio-Pliocene of the South Caribbean (Uramaco Sequence, Northwestern Venezuela). *PLoS ONE* 10:e0139230.

Cauxeiro C, Durand J, and Lopez M. 2014. Stratigraphic architecture and forcing processes of the late Neogene Miradouro da Lua sedimentary prism, Cuanza Basin, Angola. *Journal of African Earth Sciences* 95:77-92.

Clark JC, Brabb EE, Greene HG, and Ross DC. 1984. Geology of Point Reyes Peninsula and implications for San Gregorio Fault history. In: Crouch JK, and Bachman SB, eds. *Tectonics and sedimentation along the California margin*. Los Angeles, California: Pacific Section SEPM, 67-86.

Dickinson JA, and Wallace MW. 2009. Phosphate-rich deposits associated with the Mio-Pliocene unconformity in south-east Australia. *Sedimentology* 56:547-565.

Dickinson JA, Wallace MW, Holdgate GR, Gallagher SJ, and Thomas L. 2002. Origin and timing of the Miocene-Pliocene unconformity in southeast Australia. *Journal of Sedimentary Research* 72:288-303.

Domning DP. 1989. Fossil Sirenia of the West Atlantic and Caribbean Region. III. *Xenosiren yucateca*, gen. et sp. nov. *Journal of Vertebrate Paleontology* 9:429-437.

Domning DP, and Deméré TA. 1984. New material of *Hydrodamalis cuestae* (Mammalia; Dugongidae) from the Miocene and Pliocene of San Diego County, California. *Transactions of the San Diego Society of Natural History* 20:169-188.

Dorsey RJ, Housen BA, Janecke SU, Fanning CM, and Spears ALF. 2011. Stratigraphy record of basin development within the San Andreas fault system: Late Cenozoic Fish Cree-Vallecito basin, southern California. *Geological Society of America Bulletin* 123:771-793.

Dowsett HJ, and Wiggs LB. 1992. Planktonic foraminiferal assemblage of the Yorktown Formation, Virginia, USA. *Micropaleontology* 38:75-86.

Espinosa-Arrubarrena L, and Applegate SP. 1981. Selacifauna Pliocenica de Baja California Sur, Mexico y sus problemas de correlacion regional. *Anais do Congresso Latino-Americano Paleontologia* 2:667-681.

Estevens M. 2000. Miocene marine mammals from Portugal: paleogeographical and paleoecological significance. *Ciênces da Terra (UNL)* 14:323-334.

Fitzgerald EMG. 2004. A review of the Tertiary fossil Cetacea (Mammalia) localities in Australia. *Memoirs of the Museum of Victoria* 61:183-208.

Fitzgerald EMG. 2005. Pliocene marine mammals from the Whalers Bluff Formation of Portland, Victoria, Australia. *Memoirs of Museum Victoria* 62:67-89.

Fitzgerald EMG, and Kool L. 2015. The first fossil sea turtles (Testudines: Cheloniidae) from the Cenozoic of Australia. *Alcheringa* 39:142-148.

Flemming C, and McFarlane DA. 1998. New Caribbean locality for the extinct great white shark *Carcharodon megalodon*. *Caribbean Journal of Science* 34:317-318.

Franco GL, Gonzalez RA, Recio AM, Furrazola-Bermudez G, Delgado R, and Triff J. 1992. *Lexico estratigrafico de Cuba*. Havana: Ministerio de Industria Basica.

Galloway AJ. 1977. Geology of the Point Reyes Peninsula, Marin County, California. *California Division of Mines and Geology Bulletin* 202:1-72.

Garcia EXM, Antunes MT, Caceras-Balbino A, Ruiz-Munoz F, and Civis-Llovera J. 2009. Los tiburones Lamniformes (Chondrichthyes, Galeomorphii) del Pliocene inferior de la Formación Arenas de Huelva, suroeste de la cuenca del Guadalquivir, España. *Revista Mexicana de Ciencias Geologicas* 26:674-686.

González-Delgado JA, Civis J, Dabrio CJ, Goy JL, Ledesma S, Pais J, Sierro FJ, and Zazo C. 2004. Cuenca del Guadalquivir. In: Vera JA, ed. *Geología de España:*. Madrid: Sociedad Geológica Española, Instituto Geológico Minero Español, 543-550.

Hanna GD. 1926. Paleontology of Coyote Mountain, Imperial County, California. *Proceedings of the California Academy of Sciences* 24:427-503.

Hazel JE. 1971. Ostracode biostratigraphy of the Yorktown Formation (upper Miocene and lower Pliocene) of Virginia and North Carolina. . *US Geological Survey Professional Paper* 704:1-13.

Hazel JE. 1983. Age and correlation of the Yorktown (Pliocene) and Croatan (Pliocene and Pleistocene) formations at the Lee Creek Mine. *Smithsonian Contributions to Paleobiology* 53:81-199.

Holt JW, Holt EW, and Stock JM. 2000. An age constraint on Gulf of California rifting from the Santa Rosalía basin, Baja California Sur, Mexico. *Geological Society of America Bulletin* 112:540-549.

Itoigawa J, Nishimoto H, Kuroda M, Horie H, Naruse A, and Watanabe Y. 1975. *Carcharodon carcharias* (Linne) shark teeth from the Pliocene Na-arai Formation, Choshi Peninsula, environs of Tokyo, Japan. *Bulletin of the Mizunami Fossil museum* 2:91-102.

Iturralde-Vinent M, Hubbell G, and Rojas R. 1996. Catalogue of Cuban fossil Elasmobranchii (Paleocene to Pliocene) and paleogeographic implications of their lower to middle Miocene occurrence. *The Journal of the Geological Society of Jamaica* 31:7-21.

Janssen AW, Kroh A, and Avila SP. 2008. Early Pliocene heteropods and pteropods (Mollusca, Gastropoda) from Santa Maria Island (Azores, Portugal): systematics and biostratigraphic implications. *Acta Geologica Polonica* 2008:355-369.

Jordan DS, and Hannibal H. 1923. Fossil sharks and rays of the Pacific slope of North America. *Bulletin of the Southern California Academy of Science* 22:27-63.

Kanie Y, Okada H, Sasahara Y, and Tanaka H. 1991. Calcareous nannoplankton age and correlation of the Neogene Miura Group between the Miura and Boso Peninsulas, southern-central Japan. *Journal of the Geological Society of Japan* 97:135-155.

Kemp NR. 1991. Chondrichthyans in the Cretaceous and Tertiary of Australia. In: Vickers-Rich P, Monaghan JM, Baird RF, and Rich TH, eds. *Vertebrate Palaeontology of Australasia*. Melbourne: Pioneer Design Studio in cooperation with the Monash University Publications Committee.

Keyes IW. 1972. New records of the elasmobranch *C. megalodon* (Agassiz) and a review of the genus *Carcharodon* in the New Zealand fossil record. *New Zealand Journal of Geology and Geophysics* 15:228-242.

Kohno N. 1992. A new Pliocene fur seal (Carnivora: Otariidae) from the Senhata Formation on the Boso Peninsula, Japan. Natural History Research. p 15-28.

Long DJ. 1993. Late Miocene and Early Pliocene fish assemblages from the north central coast of Chile. *Tertiary Research* 14:117-126.

Longbottom AE. 1979. Miocene shark's teeth from Ecuador. *Bulletin of the British Museum (Natural History) Geology* 32:57-70.

Marsili S. 2006. Analisi sistematica, paleoecologica e paleobiogeografica della selaciofauna Plio-Pleistocenica del Mediterraneo Ph.D. Universita di Pisa.

Marsili S. 2008. Systematic, paleoecologic and paleobiogeographic analysis of the Plio-Pleistocene Mediterranean elasmobranch fauna. *Atti Della Societa Toscana Di Scienze Naturali Memorei Serie A* 113:81-88.

Martinez-Gutierrez G, and Sethi PS. 1997. Miocene-Pleistocene sediments within the San Jose del Cabo basin, Baja California Sur, Mexico. *Geological Society of America Special Paper* 318:141-166.

Marx FG, and Fordyce RE. 2015. Baleen boom and bust: a synthesis of mysticete phylogeny, diversity and disparity. *Royal Society Open Science* 2:140434.

Mazengarb C, and Speden IG. 2000. Geology of the Raukumara area. *Institute of Geological and Nuclear Sciences 1:250000 map* 6:1-60.

McKee JWA. 1987. The occurrence of the Pliocene penguin *Tereingaornis moisleyi* (Sphenisciformes: Spheniscidae) at Hawera, Taranaki, New Zealand. *New Zealand Journal of Geology and Geophysics* 14:557-561.

McKee JWA. 1994a. *Carcharodon megalodon* vertebrae from the Pliocene Tangahoe Formation, Hawera, New Zealand - with an estimation of the shark size based on these vertebrae. *Geological Society of New Zealand Miscellaneous Publication* 80A:124.

McKee JWA. 1994b. Geology and vertebrate paleontology of the Tangahoe Formation, South Taranaki coast, New Zealand. *Geological Society of New Zealand Miscellaneous Publication* 80B:63-91.

Miranda JA, Wallace MW, and McLaren S. 2009. Tectonism and eustasy across a late Miocene strandplain: the Loxton-Parilla Sands, Murray Basin, southeastern Australia. *Sedimentary Geology* 219:24-43.

Morgan GS. 1994. Miocene and Pliocene marine mammal faunas from the Bone Valley Formation of central Florida. *Proceedings of the San Diego Society of Natural History* 29:239-268.

Murakami M. 2016. A new extinct inioid (Cetacea, Odontoceti) from the upper Miocene Senhata Formation, Chiba, central Japan: the first record of Inioidea from the North Pacific Ocean. *Paleontological Research* 20:207-225.

Oishi M, and Hasegawa Y. 1995. Diversity of Pliocene mysticetes from eastern Japan. *The Island Arc* 3:346-452.

Pawellek T, Adnet S, Cappetta H, Metais E, Salem M, Brunet M, and Jaeger JJ. 2012. Discovery of an earliest Pliocene relic tropical fish fauna in a newly detected cliff section (Sabratah Basin, NW Libya). *Neues Jahrbuch für Geologie und Paläontologie Abhandlungen* 266:93-114.

Pimiento C, and Clements CF. 2014. When did *Carcharocles megalodon* become extinct? A new analysis of the fossil record. *PLoS ONE* 9:e11086.

Pledge NS. 1985. An early Pliocene shark tooth assemblage in South Australia. *Special Publications of the South Australian Department of Mines and Energy* 5:287-299.

Pomar L, Ward WC, and Green DG. 1996. Upper Miocene reef complex of the Llucamajor area, Mallorca, Spain. *SEPM Concepts in Sedimentology and Paleontology* 5:191-225.

Smith CJ, Collins LS, Jaramillo C, and Quiroz L. 2010. Marine paleoenvironment of Miocene-Pliocene formations of north-central Falcon stat, Venezuela. *Journal of Foraminiferal Research* 40:266-282.

Suzuki Y, Iryu Y, Inagaki S, Yamada T, Aizawa S, and Budd DA. 2006. Origin of atoll dolomoties distinguished by geochemiestry and crystal chemistry: Kita-daito-jima, northern Philippine Sea. *Sedimentary Geology* 183:181-202.

Takayanagi H, Nakayama Y, Ishikawa T, Nagaishi K, and Iryu Y. 2010. Sr isotope composition of island-surface dolomites at Kita-daito-jima, northern Philippine Sea. *Journal of the Geological Society of Japan* 116:237-240.

Tedford RH, Albright LB, III,, Barnosky AD, Ferrusquia-Villafranca H, R.M. Jr., Swisher CC, III,, Voorhies MR, Webb SD, and Whistler DP. 2004. Mammalian biochronology of the Arikareean through Hemphillian interval (Late Oligocene through Early Pliocene epochs). In: Woodburne MO, ed. *Late Cretaceous and Cenozoic Mammals of North America: Biostratigraphy and Geochronology*. New York: Columbia University Press, 169-231.

Tsuchi R, Shuto T, Takayama TF, A., Koizumi I, Ibaraki M, and Martinez PR. 1988. Fundamental data on Cenozoic biostratigraphy of Chile. In: Tsuchi R, ed. *Reports of Andean Studies, Shizuoka University-Trans Pacific Correlation of Cenozoic Geohistory 2*. Shizuoka: Kofune Printing, 71-95.

Turnbull WD, Lundelius EL, and McDougall I. 1965. A potassium-argon dated Pliocene marsupial fauna from Victoria, Australia. *Nature* 206:816.

Vendrasco MJ, Eernisse DJ, Powell CL, II, and Fernandez CZ. 2012. Polyplacophora (Mollusca) from the San Diego Formation: a remarkable assemblage of fossil chitons from the Pliocene of Southern California. *Los Angeles County Museum Contributions in Science* 520:15-72.

Vonk AJ, and Kamp PJJ. 2004. Late Miocene-Early Pliocene Matemateaonga Formation in eastern Taranaki Peninsula: a new 1:50,000 geological map and stratigraphic framework. *2004 New Zealand Petroleum Conference Proceedings* 7-10 March 2004:1-9.

Wade BS, Pearson PN, Berggren WA, and Pälike H. 2011. Review and revision of Cenozoic tropical planktonic foraminiferal biostratigraphy and calibration to the geomagnetic polarity and astronomical time scale. *Earth-Science Reviews* 104:111-142.

Wagner HM, Riney BO, Deméré TA, and Prothero DR. 2001. Magnetic stratigraphy and land mammal biochronology of a nonmarine facies of the Pliocene San Diego Formation, San Diego County, California. *SEPM Pacific Section Book* 91:359-368.

Walsh SA. 2001. The Bahia Inglesa Formation bonebed: genesis and palaeontology of a Neogene Konzentrat Lagerstatte from North-Central Chile Ph.D. University of Portsmouth.

Walsh SA, and Suarez ME. 2006. New penguin remains from the Pliocene of northern Chile. *Historical Biology* 18:115-126.

Watters DR, Donahue J, and Stuckenrath R. 1992. Paleoshorelines and the prehistory of Barbuda, West Indies. In: Johnson LL, ed. *Paleoshorelines and Prehistory: an investigation of method*. Boca Raton: CRC Press, 15-52.

Whitmore FC, and Barnes LG. 2008. The Herpetocetinae, a new subfamily of extinct baleen whales (Mammalia, Cetacea, Cetotheriidae). *Virginia Museum of Natural History Special Publication* 14:141-180.

Whittaker JE. 1988. *Benthic Cenozoic foraminifera from Ecuador*. London: British Museum.

Williams M, Haywood AM, Harper EM, Johnson ALA, Knowles T, Leng MJ, Lunt DJ, Okamura B, Taylor PD, and Zalasiewicz JA. 2009. Pliocene climate and seasonality in North Atlantic shelf seas. *Philosophical Transactions of the Royal Society of London* 367:85-108.

Yabe H, Goto M, and Kaneko N. 2004. Age of *Carcharocles megalodon*: a review of the stratigraphic records. *Fossils* 75:7-15.

Yabe H, and Hirayama R. 1998. Selachian fauna from the upper Miocene Senhata Formation, Boso Peninsula, Central Japan. *Natural History Research Special Issue* 5:33-61.

Yabe H, and Sugiyama T. 1935. Notes on a fossil shark's tooth found in the Daito Limestone of Kita-daito-jima, Borodino Islands. *Proceedings of the Imperial Academy* 11:149-151.

Yoshida M, Hoyanagi K, Kondo H, Inoue H, Oishi M, Yoshida H, and Yanagisawa Y. 2007. Sequence stratigraphy and organic matter preservation of the Miocene to Pliocene Tatsunokuchi Formation, Iwate, Northeast Japan. *Journal of the Sedimentological Society of Japan*. 64:21-26.
